# On the origin and evolution of SARS-CoV-2

**DOI:** 10.1038/s12276-021-00604-z

**Published:** 2021-04-16

**Authors:** Devika Singh, Soojin V. Yi

**Affiliations:** School of Biological Sciences, Georgia Institute of Technology, Atlanta, GA USA

**Keywords:** Evolutionary biology, Infectious diseases

## Abstract

The severe acute respiratory syndrome coronavirus 2 (SARS-CoV-2) is responsible for the ongoing global outbreak of a coronavirus disease (herein referred to as COVID-19). Other viruses in the same phylogenetic group have been responsible for previous regional outbreaks, including SARS and MERS. SARS-CoV-2 has a zoonotic origin, similar to the causative viruses of these previous outbreaks. The repetitive introduction of animal viruses into human populations resulting in disease outbreaks suggests that similar future epidemics are inevitable. Therefore, understanding the molecular origin and ongoing evolution of SARS-CoV-2 will provide critical insights for preparing for and preventing future outbreaks. A key feature of SARS-CoV-2 is its propensity for genetic recombination across host species boundaries. Consequently, the genome of SARS-CoV-2 harbors signatures of multiple recombination events, likely encompassing multiple species and broad geographic regions. Other regions of the SARS-CoV-2 genome show the impact of purifying selection. The spike (S) protein of SARS-CoV-2, which enables the virus to enter host cells, exhibits signatures of both purifying selection and ancestral recombination events, leading to an effective S protein capable of infecting human and many other mammalian cells. The global spread and explosive growth of the SARS-CoV-2 population (within human hosts) has contributed additional mutational variability into this genome, increasing opportunities for future recombination.

## COVID-19: How genome evolution helped spur a global pandemic

A confluence of historic gene swapping and evolutionary optimizations and adaptations helped make the coronavirus causing COVID-19 so infectious to people. Devika Singh and Soojin Yi from the Georgia Institute of Technology in Atlanta, USA, discuss the molecular evidence for ancestral recombination events and natural selection in the genome of SARS-CoV-2, the coronavirus responsible for COVID-19. In particular, the spike protein that enables viral entry into human cells shows ample signs of these evolutionary processes, which probably facilitated the virus’s spillover from other animals into humans. New variants of SARS-CoV-2 that have rapidly spread during the pandemic point to ongoing evolutionary processes, including effects of both potential functional alteration and random genetic drift. A better understanding of the virus’s evolutionary trajectory could help inform efforts to contain SARS-CoV-2 and prevent future pandemics.

## Introduction

A novel coronavirus (CoV) began to circulate among humans in Wuhan, China, around December 2019. Initially, the impact of the virus on humans was poorly understood. Since then, this virus, named “severe acute respiratory syndrome coronavirus 2” (SARS-CoV-2), has emerged as the source of a global pandemic, with nearly 115 million confirmed cases reported worldwide and over 2.56 million fatalities as of early March 2021. The pervasiveness and detrimental impact of SARS-CoV-2 across the globe has established it among the most notorious pandemics that have ever been recorded in human history.

Unfortunately, several aspects of the pandemic indicate that the current outbreak is not a singular event, nor will it be the last of its kind. First, outbreaks of coronavirus infection have occurred frequently over the last two decades, although previous episodes have remained relatively isolated at the regional level. These incidents include the first SARS outbreak in 2003 and the Middle East respiratory syndrome (MERS) in 2012, both of which induced severe human diseases^1^. Additionally, four strains of coronaviruses are known to cause milder symptoms of the common cold^2^. These incidences, coupled with the possibility that there may exist other coronavirus infections that went unrecognized, indicate that the spread of new coronaviruses among human populations is a relatively common phenomenon.

Second, these outbreaks exemplify the potential transmission of viruses from nonhuman animals to human populations. Importantly, there is ample evidence indicating that coronaviruses related to those responsible for recent epidemic outbreaks are abundant in other mammals^3–6^. Many of these viruses have the potential to infect humans^7^. The prevalence of these coronaviruses, paired with the high number of human activities involving close contact with wild mammals harboring these viruses, provides abundant opportunities for future transfers between species. In particular, SARS-related coronaviruses appear frequently in bats^8^, the likely proximal source of SARS-CoV-2 (see below). Consequently, future zoonotic transmission of coronaviruses to human populations is inevitable. Elucidating the origin and evolution of coronaviruses, as well as of other viruses, is critical to understanding the dynamics of future outbreaks and developing informed strategies to prevent subsequent global spread. In this review, we will discuss molecular evidence of the origin of SARS-CoV-2. We also discuss molecular evolutionary insights into the selective forces leading to the current pandemic, as well as the future evolutionary trajectories of SARS-CoV-2.

## Phylogenetic and genomic overview of SARS-CoV-2

**Fig. 1:**
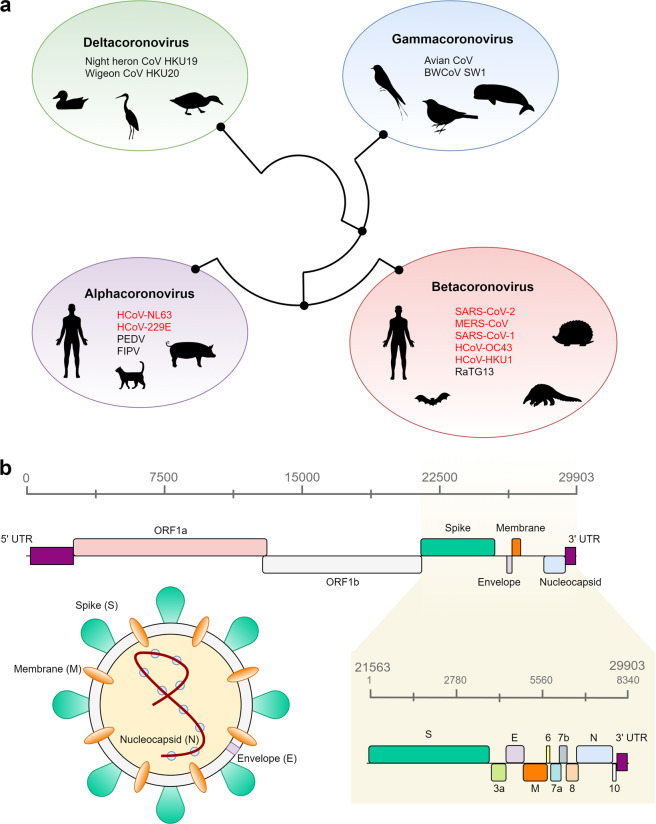
Phylogenetic background and genomic structure of SARS-CoV-2. **a** Schematic depiction of the four genera of coronaviruses, their evolutionary relationship, and their animal hosts. The phylogenetic relationships established by Woo et al.^4^ were used to draw the figure. **b** Genomic distribution of all open reading frames (ORFs) across the 29,903 bp SARS-CoV-2 genome. The nucleocapsid (N), spike (S), membrane (M), and envelope (E) proteins are color-coded according to the image of the virus. All other ORFs correspond to nonstructural proteins. The yellow panel shows an enhanced view of an 8,340 bp region encompassing 9 ORFs and the three-prime untranslated region (3′-UTR).

Coronaviruses are positive-strand RNA viruses. They are found in many animal species and may or may not cause disease symptoms in their hosts^3–6^. Based on genetic and serological characterization, coronaviruses are divided into four distinctive genera, namely, *Alphacoronavirus* (*alpha-CoV*), *Betacoronavirus* (*beta-CoV*), *Gammacoronavirus* (*gamma-CoV*), and *Deltacoronavirus* (*delta-CoV*)^3–5,9^. These groups of coronaviruses are thought to have diverged from each other at ~2400–3000 BC^4^ and tend to infect different groups of animals (Fig. 1a). Alphacoronaviruses and Betacoronaviruses are found mostly in mammals, while Gammacoronaviruses and Deltacoronaviruses are found primarily in birds, although Gammacoronaviruses also infect some cetaceans, including beluga whales and bottlenose dolphins^4,6,10,11^.

The coronaviruses that have caused the recent epidemic and pandemic outbreaks of diseases, including SARS, MERS, and COVID-19, in human populations belong to a subgroup of *Betacoronavirus* known as *Sarbecovirus*^12^. The members of this group of coronaviruses are abundant in bats and other mammals (Fig. 1a). Among the four other previously identified strains of coronaviruses associated with mild symptoms of the common cold in humans, HCoV-229E, and HCoV-NL63 belong to *Alphacoronavirus*, and HCoV-OC43 and HCoV-HKU1 are classified as a different subgroup of *Betacoronavirus* called *Embecovirus*^13^ (Fig. 1a).

Coronaviruses also undergo frequent recombination^14^. If animals harboring different coronaviruses come in close contact and exchange viruses, then recombination can occur among the different strains, leading to diversification. Unfortunately, it appears that such events during the evolutionary history of SARS-CoV-2 have led to the evolution of a potent strain capable of easily infecting human cells (see below).

Similar to other coronaviruses, SARS-CoV-2 has an ~30 kb genome^15–17^, encoding four structural proteins, including the spike protein (S), envelope protein (E), membrane protein (M), and nucleocapsid protein (N) (Fig. 1b). In addition, several nonstructural open reading frames (ORFs) are also encoded in the SARS-CoV-2 genome^16^. In infected human samples, a study found that >60% of all transcriptomes were of viral origin^16^, demonstrating the overwhelming and fundamental alteration of cell biology upon infection of human cells. Notably, these transcripts included partial transcripts, as well as noncanonical fusion transcripts^16^, as observed in previous studies of other coronaviruses^18,19^. While the functional significance of these transcripts remains unknown, their presence provides further evidence that this virus is prone to frequent recombination events within hosts.

## Evolution of SARS-CoV-2: comparisons to the closest known relative coronavirus indicate strong purifying selection and modest divergence

As of the writing of this article, one of the closest known relative of SARS-CoV-2 is a coronavirus strain found in a bat sample from Yunnan Province, China, in 2013^15^. This strain is referred to as “RaTG13” (indicating that it was found in a horseshoe bat, *Rhinolophus affinis*, in 2013). It is commonly acknowledged that the genome sequence of this strain is 96% similar to that of SARS-CoV-2^15^. At first glance, a similarity of 96% might suggest a relatively close relationship between these two viral strains. In principle, given that we have some prior knowledge on how fast coronavirus sequences accumulate nucleotide substitutions over time^4,20^, we can estimate the time to the most recent common ancestor (tMRCA) of the two strains.

Before doing so, however, we need to consider the following characteristics of coronavirus genome evolution. As shown above, the genome of SARS-CoV-2 consists nearly entirely of protein-coding sequences, a trait shared with other coronaviruses. When examining the evolution of protein-coding sequences, it is important to separately consider nucleotide substitutions that alter amino acids (and thus potentially modify the protein structure) from those that do not affect amino acids. These two types of substitutions, referred to as “nonsynonymous substitutions” and “synonymous substitutions”, respectively, are expected to evolve at different rates^21^. Nonsynonymous substitutions, because they change the underlying amino acids, can alter the functional properties of the resulting proteins. Consequently, they are likely to be subject to natural selection. In comparison, synonymous substitutions are less “visible” to natural selection because they do not affect the resulting proteins. Although some synonymous substitutions may be influenced by natural selection^22,23^, in many species, they are largely affected by underlying mutation rates, as well as by random genetic drift^24,25^.

In most genomic comparisons, nonsynonymous substitutions occur much less frequently than synonymous substitutions due to purifying natural selection, which shields the existing proteins against potentially deleterious changes in amino acid sequences. For example, in human proteins, the ratio of nonsynonymous substitutions to synonymous substitutions is on average ~0.2^26^. For the SARS-CoV-2 genome, the ratio of nonsynonymous substitutions to synonymous substitutions is 0.028 when examined across 9 ORFs in this genome^27^. There are two insights that can be gained from this observation. First, the ORFs in SARS-CoV-2 are largely maintained by purifying selection to exclude (likely deleterious) mutations. In fact, the observed ratio of nonsynonymous to synonymous substitutions is much lower than the estimates obtained from other coronaviruses^20^, indicating that the SARS-CoV-2 genome is under extremely strong purifying selection. We will discuss this further in a later section. Second, to obtain a better estimate of the time of divergence of SARS-CoV-2 from other strains, it is better to use synonymous substitutions than nonsynonymous substitutions. This is because synonymous substitutions are largely determined by the underlying mutation rates, which are likely to be more stable than selection for specific amino acid sequences. If we examine synonymous substitutions alone, the average similarity between RaTG13 and SARS-CoV-2 is only 83%, rather than 96%^27^, indicating a much more distant relationship than the initial 96% implies.

We can utilize this metric to estimate the time of divergence between SARS-CoV-2 and RaTG13^27,28^. A previous study analyzed synonymous substitutions in coronavirus genomes and estimated that synonymous substitution rates range from 1.67 to 4.67 × 10^−3^/site/year^20^. More recent studies of mutation rates in coronaviruses, including SARS-CoV-2, have provided similar estimates. For example, Li et al.^29^ and Chaw et al.^30^ estimated that the mutation rates in SARS-CoV-2 were 1.19–1.31 × 10^−3^/site/year and 1.5–3.3 × 10^−3^/site/year, respectively. Comparing these values, the divergence time between SARS-CoV-2 and RaTG13 may range from 18 to 71.4 years. Studies using more sophisticated methods for the assessment of divergence times have reported similar estimates^28^. Specifically, using a Bayesian phylogenetic approach, Boni et al.^28^ conservatively estimated that the MRCA of RaTG13 and SARS-CoV-2 likely existed in 1969 (95% highest posterior density [HPD]: 1930–2000). Given that the generation times of viruses are extremely short (in tissue cultures, SARS-CoV-2 could generate 10^3^ virions in 10 h^31^), SARS-CoV-2 and RaTG13 are, in fact, rather divergent. It is highly probable that there exist other coronaviruses that are much more closely related to SARS-CoV-2. Given that there is tremendous diversity of coronaviruses in bats and other mammals, surveillance sequencing of such coronaviruses should yield a coronavirus strain more closely related to SARS-CoV-2.

The second point to consider is that even though RaTG13 is closely related to SARS-CoV-2, there is a substantial amount of variation in sequence similarity across the genomes of these two viruses, ranging between 93.1 and 99.6% (e.g., ref. ^32^). In genome sequence comparisons, some amount of variation across genomic regions is often observed due to the underlying variation in mutation rates (e.g., refs. ^33,34^,). However, phylogenetic comparisons to other coronavirus strains and previously detected recombination events between coronavirus strains suggest that SARS-CoV-2 underwent complex recombination events during its evolution. Consequently, the evolutionary histories of different genomic segments can be distinct from each other, and different regions of the SARS-CoV-2 genome may share closer genetic divergence with coronavirus strains other than RaTG13. We will examine this observation in greater depth in the next section.

## Frequent recombination in the evolutionary history of SARS-CoV-2

Comparative analyses of coronaviruses closely related to SARS-CoV-2 have identified numerous recombination events in the evolutionary history of this virus. In fact, the genome of SARS-CoV-2 can be considered a combination of several ‘recombination blocks’ or regions between inferred breakpoints for recombination events. For example, upon comparing the genome sequences of 68 *Sarbecovirus* strains, including SARS-CoV-2, Boni et al.^28^ detected numerous recombination breakpoints in the data. The detected recombination events were found across the genome, with the highest frequency in ORF1a, followed by the region marking the N-terminus of the S protein^28^. It is important to note that even though we can detect recombination from sequence alignments, it is generally not possible to determine which genomes were ancestral to and which were the consequences of the recombination events.

Recombination events in the evolutionary history of the S protein have particular significance for the current pandemic. This protein, encoding the spike structural protein that gives coronaviruses the appearance of a “corona”, as in their namesake^35^ (Fig. 1b), is essential for the interaction with host cells. The S protein of SARS-CoV-2 binds to human angiotensin-converting enzyme 2 (ACE2) on the cell surface, allowing the virus to enter the human body^8,36,37^. Interestingly, the S proteins of coronaviruses are known to undergo frequent sequence changes in nature, including deletions, mutations, and recombination^14^. Notably, the receptor-binding domain (RBD) of the spike protein of SARS-CoV-2 shows more divergence from the RaTG13 strain than other regions, suggesting some alteration in the binding affinity to human ACE2^37^. Overall, the evolutionary history of the S protein of SARS-CoV-2 appears to be highly complex; the entirety of the S protein sequence consists of several segments with different phylogenetic relationships among the examined *Sarbecovirus* strains^28^.

**Fig. 2:**
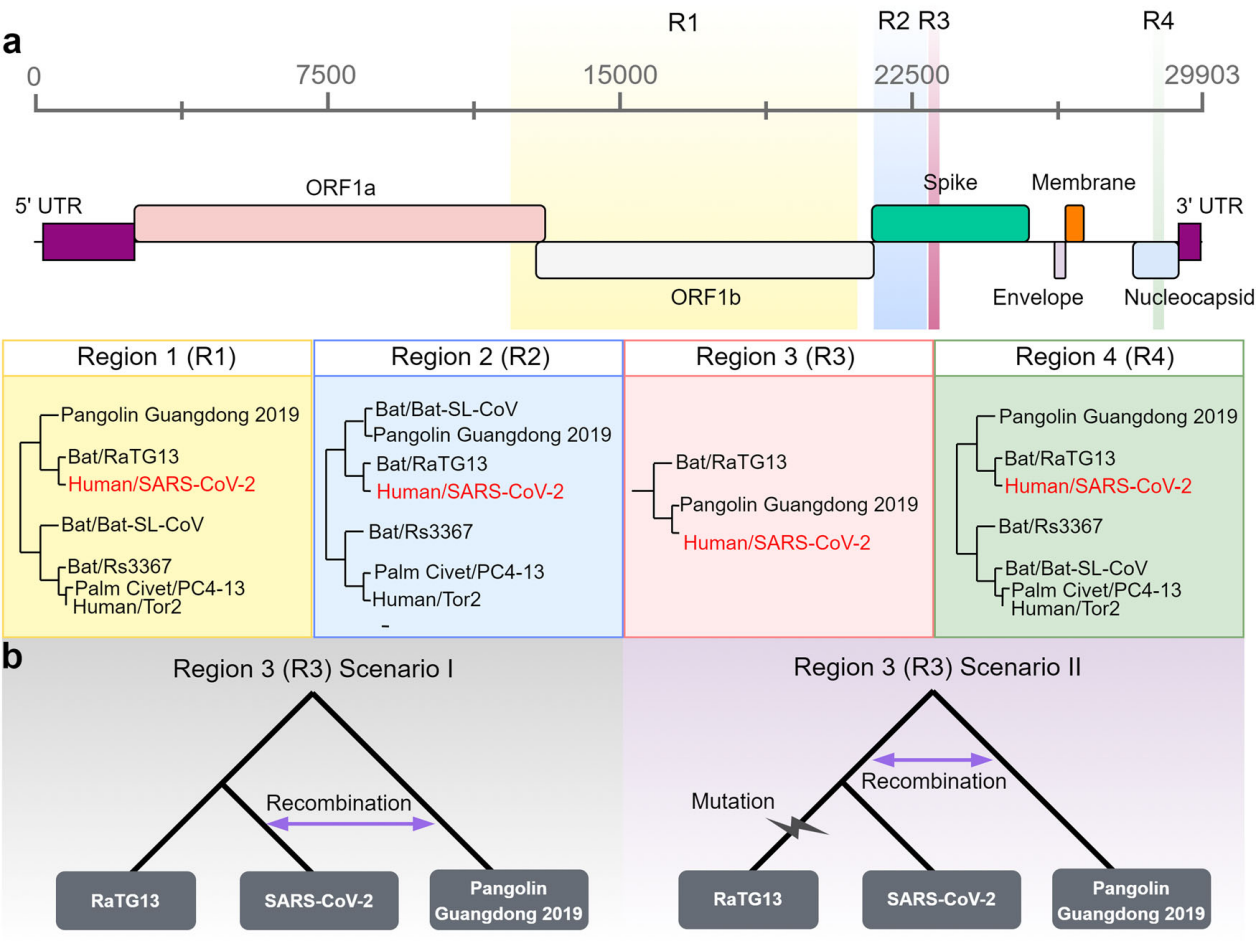
Recombination events in the history of SARS-CoV-2. **a** Variations in the sequence relatedness of different regions of SARS-CoV-2 in comparison with alternative strains of coronaviruses from pangolins (Pangolin Guangdong 2019), bats (RaTG13, Bat-SL-CoV, Rs3367), palm civets (PC4-13), and humans (Tor2). In region 1 (R1), region 2 (R2), and region 4 (R4), SARS-CoV-2 is most similar to the corresponding regions of the bat coronavirus RaTG13. In region 3 (R3), SARS-CoV-2 and the pangolin strain of coronavirus (Pangolin Guangdong 2019) are more closely related. The pangolin strain consistently clusters within bat coronavirus clades. For regions 1, 2, and 3, phylogenetic relationships were obtained from Lam et al.^32^; for region 4, phylogenetic relationships were obtained from Boni et al.^28^. Regions are colored based on their genomic position in the SARS-CoV-2 genome model (top panel). **b** Two scenarios hypothesizing the evolutionary timing of the recombination event that may have introduced the pangolin coronavirus sequence (region 3/R3) into SARS-CoV-2. In scenario I, after the divergence of SARS-CoV-2 and RaTG13, recombination between SARS-CoV-2 and Pangolin Guangdong 2019 resulted in the acquisition of the new sequence. In scenario II, recombination occurred between the common ancestral lineage of SARS-CoV-2 and RaTG13 and the pangolin (Pangolin Guangdong 2019) lineage, followed by the accumulation of mutations in the RaTG13 lineage.

A representative subset of these recombination blocks is illustrated in Fig. 2a (denoted as four “regions” in sequential order across the SARS-CoV-2 genome). The first region (R1 in Fig. 2a) spans ORF1b, and the second region (R2 in Fig. 2a) encompasses the 5′ region of the S protein. Another representative recombination block is observed in the nucleocapsid protein (R4 in Fig. 2). The phylogenies within R1, R2, and R4 indicate that SARS-CoV-2 is most similar to the corresponding regions of the bat coronavirus RaTG13 (Fig. 2a). Interestingly, in all three regions, a coronavirus sampled from a pangolin in 2019 (referred to as “Pangolin Guangdong 2019” in Fig. 2, following Boni et al.^28^) shows a close phylogenetic relationship to the common ancestor of SARS-CoV-2 and RaTG13 (Fig. 2a^32^). Region 3 (R3) in Fig. 2a includes the variable loop region of the SARS-CoV-2 S protein, which is 222 bp in length and contains 6 residues of the RBD. Remarkably, this region shares the closest similarity with the pangolin coronavirus strain, rather than with RaTG13^28,32^ (Fig. 2a).

Thus, the variable loop region of the S protein shows a unique evolutionary history compared to the rest of the S protein and the SARS-CoV-2 genome overall, consistent with two different scenarios (Fig. 2b): first, after the lineages leading to the SARS-CoV-2 and RaTG13 split, a recombination event between the SARS-CoV-2 lineage and the Pangolin Guangdong 2019 lineage resulted in the acquisition of new RBD residues (Fig. 2b, left panel). A second scenario is that recombination with the Pangolin Guangdong 2019 lineage occurred in the common ancestral lineage of SARS-CoV-2 and RaTG13. Subsequently, the RaTG13 lineage accumulated additional sequence variation due to mutations and/or other events, thus becoming more distantly related to SARS-CoV-2 (Fig. 2b, right panel). It is notable that outside of the variable loop, RaTG13 was still the closest relative to SARS-CoV-2 (Fig. 2a). In contrast, Pangolin Guangdong 2019 was more distantly related to SARS-CoV-2 than RaTG13 for most of the S protein as well as the rest of the genome^28^.

**Fig. 3:**
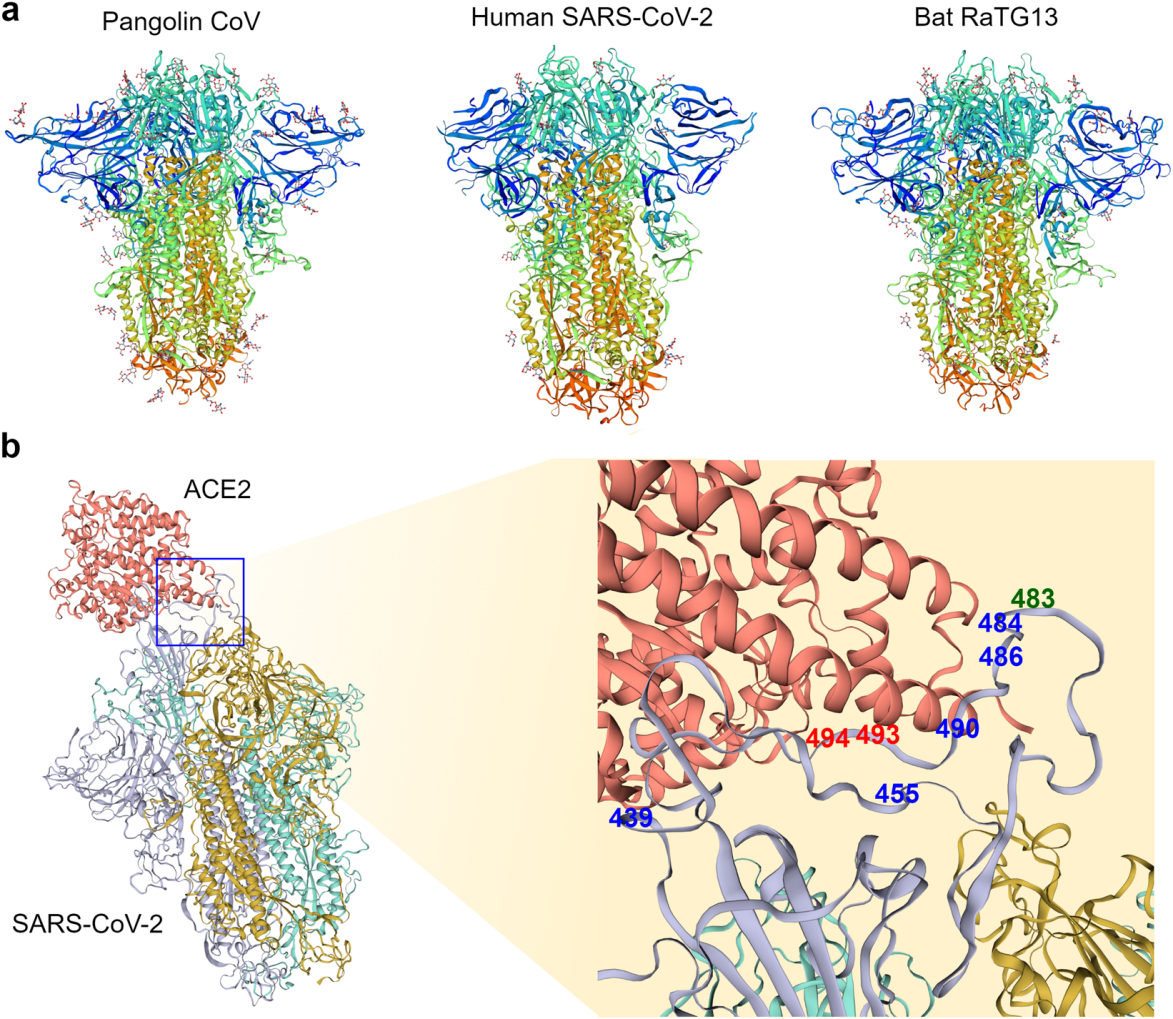
Structural comparison of coronavirus spike proteins. **a** Long-axis trimer, closed conformation view of the cryo-EM spike protein structure from the pangolin coronavirus (PDB ID: 7CN8, left panel), human SARS-CoV-2 (PDB ID: 6ZB5, middle panel), and bat RaTG13 coronavirus (PDB ID: 7CN4, right panel). Models are rainbow colored from the N-terminus (blue) to the C-terminus (red). **b** Left panel depicts human SARS-CoV-2 (colored by chain: purple, yellow, and green) and the ACE2 complex (colored red) in bound confirmation (PDB ID: 6ACG). The right panel shows a magnified region encompassing 8 amino acids (positions shown in blue, green, and red) detected as targets of positive selection in the previous studies^27,47,50^. The positions in blue were identified as positively selected in one of the three cited studies, while the positions depicted in red (493 and 494) were identified in two studies. The green position 483 of the S protein was identified as positively selected in all three studies. For **a**, **b** all models were visualized by SWISS-MODEL^66^.

Pangolin Guangdong 2019 (from Guangdong) and other coronaviruses from pangolins (sampled in 2017 from Guangxi) are phylogenetically located within the same clade as coronaviruses from bats^28,32,38^. The cryo-EM structures of coronaviruses from pangolins and bats are also similar to each other^39,40^ (Fig. 3a). Consequently, it was proposed that the Malayan pangolins (*Manis javanica*) may have been an intermediate host of SARS-CoV-2^32,38^. It should be stated that the pangolins from which these viruses were obtained were rescued from illegal smuggling operations and that efforts to identify coronaviruses from wild pangolins were unsuccessful^41^. Therefore, the conclusion that wild pangolins serve as a direct intermediary host is still under debate. It is possible that there was an ancestral recombination event between the lineages leading to the pangolin-CoV and SARS-CoV-2 that took place in bats or in another intermediate host. At the very least, the presence of multiple different phylogenetic patterns indicates that coinfection and genetic recombination of coronaviruses from distantly related mammals have occurred in the recent evolutionary history of SARS-CoV-2. Even though we cannot state with confidence whether the transmissions between species occur by direct transmission or via an intermediate host, we can use these findings to propose practical and useful implications to inform strategies for working in close proximity with wild mammals to avoid future outbreaks due to continuously evolving recombinants.

## Comparative analyses of natural selection in the SARS-CoV-2 genome

SARS-CoV-2 has infected global human populations with astonishing efficiency, giving rise to some compelling questions. For example, was this pandemic fueled by the adaptive evolution of SARS-CoV-2? To explore this topic, recent work has focused on the molecular evolution of the S protein, which binds human ACE2 and thus facilitates infection in the human body^42–45^. In contrast to the S protein of other bat coronaviruses, the S protein of SARS-CoV-2 is particularly efficient at binding human ACE2 and thus promotes the rapid spread of the virus throughout global populations^46,47^. It would be fascinating to investigate whether the molecular properties of the S protein that allow it to bind to human ACE2 were driven by adaptive evolution (also referred to as positive selection) in SARS-CoV-2 prior to entering the human population.

Comparing genomic sequences of different coronaviruses can provide clues to the presence and target regions of positive selection during the evolutionary histories of the viruses of interest. One of the most widely used methods involves examining the ratio of nonsynonymous to synonymous substitutions, often referred to as “dN/dS” or “ω”. A small ω indicates the occurrence of purifying selection to remove deleterious new mutations. On the other hand, this value increases if positive selection facilitates the maintenance of nonsynonymous substitution(s). Analyses of dN/dS, in a framework of log-likelihood tests, are powerful methods to detect positive selection^48^. Tang et al.^27^ compared the sequences of 13 ORFs from SARS-CoV-2 with those of several other closely related coronaviruses, including those from bats, pangolins, and SARS-CoV, which caused the previous SARS epidemic in 2002–2003. They found that in all pairwise comparisons, the ω values of the examined ORFs ranged between 0.044 and 0.124. Thus, all SARS-CoV-2 ORFs exhibit signs of strong purifying selection, rather than positive selection, compared to the ORFs of these other viruses.

Even though the overarching genomic trend indicates strong purifying selection, adaptive changes in amino acids can be highly localized to specific positions and/or to specific lineages. When Tang et al.^27^ further examined whether signatures of positive selection for specific amino acid positions exist, they found that their data were better explained by a model including positive selection on some positions than by one without any positive selection^27^. In their analysis of 9 ORFs, they detected 10 nonsynonymous positions that may have been subjected to positive selection. Interestingly, 3 of the 10 positions were found in the S protein, in or around the RBD (Fig. 3b).

It is important to note that the results of this type of analysis are influenced by the genome sequences used. For example, in another analysis with different coronavirus strains, Damas et al.^47^ also identified three positively selected positions in the RBD of the S protein, although only one of them overlapped with the positively selected sites identified by Tang et al.^27^. On the other hand, Li et al.^49^ concluded that strong purifying selection and recombination explained the molecular evolution of the S protein. More nuanced insights can be gained when within-population variation is considered. For example, Cagliani et al^50^. used a method that combines divergence between strains exhibiting within-population variation. They compared 44 SARS-CoV-2 genome sequences to the RaTG13 genome. Their results also suggested that while the majority of the SARS-CoV-2 genome is evolving under purifying selection, there exists evidence of positive selection for 7 positions, including 6 positions in the S protein^50^. Two of these 6 positions were also identified in earlier studies (Fig. 3b). Interestingly, some of these positions were shown to favor interactions with the ACE2 receptor in experimental and modeling studies^44,51^.

Natural selection may act on features of the SARS-CoV-2 genome independent of its proteins. Berrio et al.^52^ examined the genomes of SARS-CoV-2 and 6 other *Sarbecoviruses* to detect excess nucleotide substitutions independent of whether they occur at nonsynonymous or synonymous sites. They reasoned that this method could detect signatures of positive selection (in the form of more nucleotide substitution than expected by random chance) that are not necessarily associated with amino acid sequences. For example, the stability of the negative RNA template used for replication of the positive-strand RNA genome could be a target of natural selection: a more stable RNA molecular structure could be favored, allowing rapid and stable replication of the positive-strand viral genome. Berrio et al.^52^ found several genomic regions bearing signatures of positive selection, including some sites on the S protein (but not in the RBD).

In summary, comparative analyses of SARS-CoV-2 and other coronaviruses found consistent global signals of purifying selection, with some evidence of site-specific positive selection, especially on the S protein^27,47,50,53^. These studies provide useful targets of future studies to determine the functional significance of molecular evolutionary signatures of the SARS-CoV-2 genome (Fig. 3b). At the same time, it should be noted that the specific targets of inferred positive selection varied between studies. This could be potentially due to the difference between specific datasets analyzed. Another important caveat is that tests of positive selection can suffer from substantial rates of false positives in regions experiencing high recombination^54^. Put simply, recombination events during the evolutionary history of the genome of interest would introduce new amino acid sequences, confounding the inference of positive selection. Considering that there is a convincing body of evidence indicating that the SARS-CoV-2 genome has experienced frequent recombination, the results of comparative positive selection analyses should be taken with caution until we have a more definitive understanding of the evolutionary history of SARS-CoV-2.

## Ongoing evolution of SARS-CoV-2

Given that the virus has now been circulating in the human population for over a year and has experienced explosive population growth by infecting tens of millions of humans, another critical question emerges from the ongoing evolutionary trajectory of SARS-CoV-2. Does the SARS-CoV-2 genome show any evidence that it is adapting to human hosts during the course of the pandemic? Insights into this question can be gained from population genetic analyses of SARS-CoV-2 genomes. Indeed, the global effort to understand the dynamics of SARS-CoV-2 continues to generate such data (currently, there are more than 600,000 related datasets and counting, based on the Global Initiative on Sharing All Influenza Data (GISAID) repertoire and the Nextstrain database). With the availability of large SARS-CoV-2 genome sequence datasets, scientists can monitor the evolution of SARS-CoV-2 from its initial introduction to the human population and determine if any specific sites show evidence of adaptive evolution. Such studies will help us understand the dynamics of coronavirus spread and its impact on public health while also aiding in identifying targets and candidates for vaccines and therapeutic interventions. Undoubtedly, this is an extremely active area of ongoing research, with many more insights to be gained in the near future, potentially in the coming months.

**Fig. 4:**
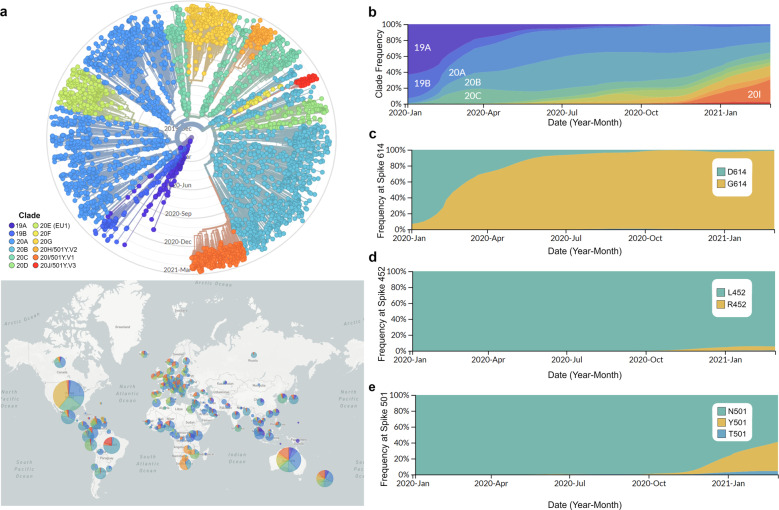
Demographics of SARS-CoV-2. **a** Top panel shows a phylogenetic tree of 3852 SARS-CoV-2 genomes sampled globally between December 2019 and March 2021. The bottom panel shows the geographic distribution of the major clades of SARS-CoV-2. Clades were defined using Nextstrain nomenclature based on global frequency, variation from parent clade, and year of emergence. The relative global frequency of **b** all major SARS-CoV-2 clades, **c** the amino acid variant at position 614 of the spike protein (D: aspartic acid and G: glycine), **d** the amino acid variant at position 452 of the spike protein (L: leucine and R: arginine), and **e** the amino acid variant at position 501 of the spike protein (N: asparagine, Y: tyrosine and T: threonine). For **a**, **b** clades were named according to Nextstrain nomenclature, which distinguishes clades based on global frequency, year of emergence and a unique letter. For **a**–**e** data visualization was performed by nextstrain.org with data provided by GISAID.

Analyses of globally sampled SARS-CoV-2 genomes during the course of the pandemic have revealed the presence of several subgroups of the virus harboring distinctive mutations. SARS-CoV-2 genomes appear to evolve relatively slowly, and the origin of SARS-CoV-2 is estimated as between October 2019 and December 2019 (ref. ^55^ and references therein). To date, at least 12 major lineages of SARS-CoV-2 have been identified, each with several distinguishing single-nucleotide polymorphisms (SNPs)^55–58^ (Fig. 4a, b). Inferring the functional consequences of these SNPs has tremendous implications for understanding the future trajectories of the pandemic and developing preventive measures and treatment strategies.

Interestingly, some of these mutations show steady and rapid increases in frequency during the current pandemic. In particular, much attention has been given to SNPs that change the sequence of the S protein which have the potential to alter the efficiency of the viral entry into human cells. One such mutation has sparked significant discussion. Specifically, an A at position 23,403 of the reference genome of SARS-CoV-2 is mutated to a G, resulting in an amino acid change from an aspartate [D] to a glycine [G] at residue 614. Thus, this SNP is commonly referred to as “D614G” or “G614” when referring to the derived strain in comparison to the ancestral “D614”. Remarkably, the frequency of the viruses harboring the G614 mutation (referred to as the G614 variant) has increased worldwide since the beginning of the pandemic, and it has now become the dominant global strain^56,58^ (Fig. 4c). Molecular dating indicates that this novel mutation arose early in the pandemic^58^. The rapid and global increase in this variant led some researchers to hypothesize that it was caused by a selective advantage^56^. However, it is important to note that genetic drift can also cause an increase in the frequency of a specific variant without selective advantage, especially if the mutation occurred early in the growing population.

Determining whether this mutation confers a selective advantage to SARS-CoV-2 in its infection of human populations is an ongoing topic of research with no definitive conclusion thus far. Protein structure analyses by cryogenic electron microscopy and computation modeling were unable to provide evidence that this mutation would significantly increase the interaction between the S protein and ACE2^58,59^. On the other hand, this variant was associated with increased viral loads in COVID-19 patients^56^. However, viral loads may depend on other variables, such as genetic and environmental factors specific to patients. In addition, it is difficult to definitively identify independent effects of D614G because the D614G mutation cooccurs with other SNPs in linkage disequilibrium. In additional studies, scientists engineered D614 and G614 mutations in other viruses^60^ and in the reference SARS-CoV-2 strain^61^ in an attempt to directly measure functional effects. Even in such settings, elucidating the selective advantage or disadvantage of a specific mutation is challenging, given the complexity of viral infections and the immune response. Nevertheless, the immense potential of such functional studies to disentangle the effects of different mutations arising in the SARS-CoV-2 population would provide insights with tremendous implications for treating COVID-19 patients and devising public health strategies.

Despite the low mutation rate, the extremely large population size and prolonged duration of the pandemic have fueled the introduction of new variants into SARS-CoV-2 genomes worldwide. In addition, the rapid population growth of SARS-CoV-2 can facilitate the enrichment of specific mutations via founder effects. We show the dynamics of SARS-CoV-2 variants harboring mutations at two additional positions of the S protein in Fig. 4d, e. One variant, containing an amino acid change from a leucine [L] to an arginine [R]) at residue 452 (L452R), is a dominant variant found in California, USA, in January 2021^62^. Other positions, such as the asparagine [N] at residue 501, have been found to harbor different mutations in different SARS-CoV-2 lineages, and at least two variants at this position (N501T and N501Y) are currently circulating (Fig. 4e)^63,64^.

Therefore, the S protein of SARS-CoV-2 continues to diversify due to its propensity for mutation and recombination. In addition, widespread infection among humans is now posing additional threats to other mammals that interact with human populations, as secondary and even tertiary transmissions between humans and other mammals can occur^65^. Such cross-specific transmission can further enable the emergence of potentially dangerous recombinant SARS-CoV-2 strains. It is imperative that epidemiological, genetic, and functional studies of variants be fully utilized to determine how to slow down and ultimately eradicate within- and between-species transmissions.

## Conclusions

Molecular evolutionary analyses of the SARS-CoV-2 reference genome indicate that SARS-CoV-2 originated from virus reservoirs in nonhuman mammals, such as bats, through recombination and purifying selection. These observations suggest that transmission events of coronaviruses between mammals, including humans, can occur and that coronavirus genomes can accumulate new variants via recombination between divergent strains residing in different host species. The COVID-19 pandemic has facilitated scientific communication and data sharing in the public domain, enabling scientists to trace recombination and transmission events occurring in the SARS-CoV-2 population in real time. The current pandemic, rapid accumulation of data, and explosive scientific analyses provide ample opportunities to develop gold standard science-guided policies for the design and implementation of epidemiological practices to prevent future outbreaks.
